# Cognitive training for children with ADHD: a randomized controlled trial of cogmed working memory training and ‘paying attention in class’

**DOI:** 10.3389/fpsyg.2015.01081

**Published:** 2015-07-28

**Authors:** Marthe van der Donk, Anne-Claire Hiemstra-Beernink, Ariane Tjeenk-Kalff, Aryan van der Leij, Ramón Lindauer

**Affiliations:** ^1^Academic Medical Center, Department of Child and Adolescent Psychiatry, University of AmsterdamAmsterdam, Netherlands; ^2^De Bascule, Academic Center for Child and Adolescent PsychiatryDuivendrecht, Netherlands; ^3^Research Institute of Child Development and Education, University of AmsterdamAmsterdam, Netherlands

**Keywords:** ADHD, school-aged children, cognitive training, academic performance, randomized controlled trial

## Abstract

The goal of this randomized controlled trial was to replicate and extend previous studies of Cogmed Working Memory Training (CWMT) in children with Attention-deficit/hyperactivity disorder (ADHD). While a large proportion of children with ADHD suffer from academic difficulties, only few previous efficacy studies have taken into account long term academic outcome measures. So far, results regarding academic outcome measures have been inconsistent. Hundred and two children with ADHD between the age of 8 and 12 years (both medicated and medication naïve) participated in current randomized controlled trial. Children were randomly assigned to CWMT or a new active combined working memory- and executive function compensatory training called ‘Paying Attention in Class.’ Primary outcome measures were neurocognitive functioning and academic performance. Secondary outcome measures contained ratings of behavior in class, behavior problems, and quality of life. Assessment took place before, directly after and 6 months after treatment. Results showed only one replicated treatment effect on visual spatial working memory in favor of CWMT. Effects of time were found for broad neurocognitive measures, supported by parent and teacher ratings. However, no treatment or time effects were found for the measures of academic performance, behavior in class or quality of life. We suggest that methodological and non-specific treatment factors should be taken into account when interpreting current findings. Future trials with well-blinded measures and a third ‘no treatment’ control group are needed before cognitive training can be supported as an evidence-based treatment of ADHD. Future research should put more effort into investigating why, how and for whom cognitive training is effective as this would also potentially lead to improved intervention- and study designs.

## Introduction

Attention-deficit/hyperactivity disorder (ADHD) is a developmental psychiatric disorder that has its onset in early childhood and is characterized by inattention, impulsivity, and/or hyperactivity (American Psychiatric Association [APA], 2000). Multimodal treatment approaches, for instance psycho stimulant medication in combination with behavioral treatment, are recommended (Taylor et al., 2004). Despite the fact that this multimodal approach has been shown to be effective in reducing ADHD symptoms (MTA Cooperative Group, 1999; Van der Oord et al., 2008), it seems that these effects cannot be sustained beyond 24 months (Jensen et al., 2007). Furthermore in regard to stimulant medication, some children experience serious side effects (Graham and Coghill, 2008) and there is growing concern among parents about the unknown long term effects (Berger et al., 2008). Finally, it has been shown that current multimodal approach does not lead to improvements in academic performance (Raggi and Chronis, 2006; Van der Oord et al., 2008), a key area of functioning in every day life which is often disturbed in children with ADHD (Loe and Feldman, 2007). These limitations have led to a growing demand for alternative non-pharmacological interventions for children with ADHD.

Of great interests are interventions that target the underlying cognitive deficits which are assumed to mediate ADHD causal pathways. Targeting those underlying cognitive deficits would potentially lead to greater transfer and generalization to functioning in every day life (Sonuga-Barke et al., 2014). Within the domain of cognitive interventions, working memory (WM) training has received most attention as a potential effective intervention for children with ADHD for several reasons. First of all, WM (i.e., the function of actively holding in mind and manipulating information relevant to a goal) is a necessary mechanism for many other complex tasks such as learning, comprehension, and reasoning (Baddeley, 2007). Second, it is assumed that WM deficits are part of the causal pathway to ADHD symptoms (Barkley, 1997; Willcutt et al., 2005). It is estimated that 81% of children with ADHD have a deficit in the *working* component (central executive) of WM (Rapport et al., 2013), in contrast to the less impaired *memory* component (phonological and visuospatial storage/rehearsal).

One of the most widely implemented and investigated interventions that targets WM is Cogmed Working Memory Training (CWMT). The rationale behind this training is that by adaptively and intensively training both the storage and storage plus manipulation components of WM, improvements will transfer to other cognitive functions such as attention as a function of underlying overlapping neural networks (Klingberg, 2010). So far, nine studies (Klingberg et al., 2002, 2005; Holmes et al., 2010; Gray et al., 2012; Green et al., 2012; Egeland et al., 2013; Hovik et al., 2013; Chacko et al., 2014; van Dongen-Boomsma et al., 2014) that investigated the efficacy of CWMT in children with ADHD reported neurocognitive outcome measures. Six of these studies showed treatment effects on trained WM tasks (Klingberg et al., 2002, 2005; Gray et al., 2012; Green et al., 2012; Hovik et al., 2013; Chacko et al., 2014) and two studies have also shown treatment effects on untrained WM tasks (Holmes et al., 2010; Hovik et al., 2013). Within the literature this latter often refers to near transfer, i.e., improvement in untrained tasks that rely on identical cognitive processes that are targeted by the intervention. Furthermore, treatment effects have also been found on measures of attention (Klingberg et al., 2002, 2005), parent ratings of ADHD related behavior (Klingberg et al., 2005; Beck et al., 2010) and parent ratings of executive functioning (Beck et al., 2010). It has been suggested (e.g., Klingberg, 2010) that this should be interpreted as evidence for far transfer, i.e., improvements in tasks that tap cognitive processes other than the trained process. Despite these promising results, there are several meta-analyses (Melby-Lervåg and Hulme, 2013; Rapport et al., 2013; Cortese et al., 2015) that are skeptical about the putative effects of WM interventions such as CWMT, mainly regarding the far transfer measures such as academic performance.

Interestingly, within the scope of CWMT efficacy studies in children with ADHD, only few have also taken into account academic outcome measures (Gray et al., 2012; Green et al., 2012; Egeland et al., 2013; Chacko et al., 2014). This is remarkable both from a scientific and clinical perspective, as interventions that can alleviate the encountered academic problems for children with ADHD are needed. Up till now, studies that did investigate the effects on academic performance found treatment effects on off task behavior (Green et al., 2012) and reading (Egeland et al., 2013). Despite these promising results and on the other hand the critical notes from previous meta-analyses (Melby-Lervåg and Hulme, 2013; Rapport et al., 2013; Cortese et al., 2015), we do suggest that replication of previous CWMT studies in children with ADHD is necessary. There is still no consistent pattern of results, mainly in regard to far transfer measures such as academic performance. It has been noted that previous effect studies suffered from both theoretical and methodological flaws and several suggestions have been made to optimize future research.

The most frequently addressed methodological issue concerns the use of an inadequate control group (Shipstead et al., 2010, 2012a,b; Morrison and Chein, 2011; Chacko et al., 2013; Melby-Lervåg and Hulme, 2013). Within the scope of CWMT effect studies in children with ADHD, some studies have used non-active (e.g., waiting list, treatment as usual) control groups (Beck et al., 2010; Egeland et al., 2013; Hovik et al., 2013) which hinders blinding (Sonuga-Barke et al., 2014) and only overcomes simple test–retest effects (Morrison and Chein, 2011; Shipstead et al., 2012b). Others (Klingberg et al., 2002, 2005; Green et al., 2012; van Dongen-Boomsma et al., 2014) used low-demand, non-adaptive placebo versions which require considerably less time and effort then the active condition which also diminishes the amount and quality of interaction with the training aide (most often a parent) and CWMT coach (Chacko et al., 2013). Furthermore, in regard to academic outcome measures in previous CWMT studies in children with ADHD, only the study of Egeland et al. (2013) included long term assessment. Gathercole (2014) recently suggested that long term assessment of standardized academic ability tests are crucial as the child will need to exploit his or her improved WM capacity and this will only be visible after a lengthy period. Others (Sonuga-Barke et al., 2014; Cortese et al., 2015) also suggested that future trials should include a broader range of functional outcomes and long-term follow-up.

In current study we will replicate and, moreover, extend previous CWMT studies in children with ADHD between the age of 8 and 12 years by investigating the effects on neurocognitive functioning, academic performance, behavior in class, behavior problems and quality of life. As has been suggested (Shipstead et al., 2010, 2012a,b; Morrison and Chein, 2011; Chacko et al., 2013; Melby-Lervåg and Hulme, 2013), we will compare these effects with an active control group whose experience is closely matched to the training group in terms of effort (adaptive WM tasks in response to performance), time (equal interaction time with the coach) and performance related feedback. This active control group receives a cognitive training called ‘Paying Attention in Class’ (PAC) which was developed by the authors. This training consists of a WM – and a compensatory executive function training. Next to adaptive WM tasks, this intervention also targets a broader set of executive functions that are impaired in children with ADHD with a main focus on how to use those executive functions in the classroom. The following research questions were addressed in this study: (1) What are the effects of CWMT on measures of neurocognitive functioning, academic performance, behavior in class, behavior problems and quality of life? and (2) Is an active control intervention equally effective as CWMT?

## Materials and Methods

### Participants

Children were recruited in two different ways for this study. First, clinical care providers from two clinical care departments of the De Bascule (Academic Centre for Child and Adolescent Psychiatry, Amsterdam) referred eligible children to the researcher. Second, healthcare staff members (usually remedial teacher or school psychologist) of schools in the region of Amsterdam contacted the researcher when they had eligible children. In both cases, the researcher visited the school for an information meeting to extensively inform the staff members. Parents of children who met criteria for participation were approached and informed by the school staff member. Eligible participants were (a) children between the age of 8 and 12 years, (b) diagnosed with ADHD by a professional according to the guidelines of the Diagnostic and Statistic Manual of Mental Disorders DSM-IV (American Psychiatric Association [APA], 2000). Children with comorbid learning disabilities (LDs) and/or oppositional defiant disorder (ODD) were also included. Children on medication were only included when they were well-adjusted to their medication, which meant that they were not participating in a medication trial, and type and dosage of medication was unchanged at least 4 weeks prior to the start and during the training. Exclusion criteria were (a) presence of psychiatric diagnoses other than ADHD/LD/ODD, (b) Total Intelligence quotient < 80, (c) significant problems in the use of the Dutch language and (d) severe sensory disabilities (hearing/vision problems). Parents filled out an application package containing a written informed consent form, questionnaires of demographic- and background information and the Dutch translation of the Social Communication Questionnaire (SCQ; Warreyn et al., 2004) to screen for autism spectrum disorder. The ‘Lifetime’ version of the SCQ consists of 40 questions that have to be answered with ‘yes’ or ‘no.’ A total raw score of 15 or higher indicates a likelihood of the presence of autism spectrum disorder and is recommended as a cutoff-score. Children with a total score of 15 or higher were excluded from this study. The attention/hyperactivity, ODD and Conduct Disorder modules of the Diagnostic Interview Schedule for Children IV (DISC-IV; Steenhuis et al., 2009) were administered by the research assistant(s) by telephone to confirm ADHD diagnose and to rule out for potential Conduct Disorder. Parents were also asked to send a copy of the diagnostic psychiatric report of their child to establish the subtype of ADHD and rule out other potential psychiatric problems that met exclusion criteria. The expert view, based on the diagnostic psychiatric report, was leading for establishing the subtype of ADHD. If the subtype was not described in the report, we used the Attention/Hyperactivity module of the DISC-IV (Steenhuis et al., 2009) to establish the subtype. A short version of the WISC-III-nl (Wechsler, 2005) with the subtests Similarities, Block Design, Vocabulary and Information was administered to estimate the Total Intelligence quotient if there were no prior recordings available. At baseline, there were no significant differences between the two groups for the demographical and clinical characteristics (**Table 1**) except for type of education. The PAC group contained significantly more children from special primary schools (e.g., children with mild learning- or behavior difficulties) but no children from special education schools (e.g., children with severe behavior or psychiatric problems).

**Table 1:** Demographic and clinical characteristics.

	CWMT (*n* = 50)	PAC (*n* = 50)	*p (t, X^2^, or Fisher’s exact test)*
Age, mean (*SD*) in years	9.8 (1.3)	10.0 (1.3)	ns
Gender			
Male, no (%)	35 (70)	37 (74)	ns
Full-Scale IQ, mean (*SD*)	103.1 (15.1)	99.2 (12.9)	ns
Medication for ADHD, no (%)	26 (55.3)	29 (61.7)	ns
ADHD diagnose, no (%)			
Combined	29 (58)	35 (70)	ns
Inattentive	15 (30)	10 (20)	
Not otherwise specified	6 (12)	5 (10)	
Comorbid disorders, No (%)			
Dyslexia	8 (21.1)	15 (35.7)	ns
Dyscalculia	0	2 (4.8)	
Oppositional defiant disorder	2 (5.3)	0	
Enrollment, no (%)			
Clinical care	7 (14)	14 (28)	ns
School	43 (86)	36 (72)	
Type of education, no (%)			
Regular primary	44 (88)	43 (86)	*X*^2^(2) = 6.789, *p =* 0.034
Special primary	2 (4)	7 (14)	
Special education	4 (8)	0	
SES, no (%)			
Low < 25.000	10 (24.4)	6 (13.6)	ns
Average 25.00–35.000	6 (14.6)	12 (27.3)	
High > 35.000	25 (61)	26 (59.1)	
Ethnicity, no (%)			
Mother Dutch	41 (87.2)	36 (73.5)	ns
Father Dutch	35 (76.1)	31 (63.3)	ns

*CWMT, Cogmed Working Memory Training; PAC, Paying Attention in Class; SES, social economic status.*

### Interventions

#### Cogmed Working Memory Training

Cogmed Working Memory Training is a computerized training program aimed to train WM. It consists of a variety of game-format tasks that are adaptive, which means that difficulty level is being adjusted automatically to match the WM span of the child on each task. The program includes 12 different visuospatial and/or verbal WM tasks, eight of these tasks (90 trials in total) are being completed every day (Klingberg et al., 2005). Children followed the standard CWMT protocol which means following the computer training program for 5 weeks, five times a week, ∼45 min a day. The program was provided via the internet on a laptop in a separate room. Children were trained individually at school, guided by a trained developmental psychologist (training aid) who was supervised by a certified Cogmed Coach. Teachers were invited to attend an information meeting in which the content of CWMT was explained by first author, it was communicated that teachers did not have an active role during treatment if children received CWMT.

#### Paying Attention in Class

‘Paying Attention in Class’ is an experimental combined WM- and compensatory training that has been developed by members of our research team. Children are trained individually outside the classroom for 5 weeks, five times a week, ∼45 min a day; the same duration as in the CWMT protocol. This PAC intervention contains three key elements; first of all, this intervention offers psycho education about executive functions that are related to classroom behavior. By making children more aware of these executive functions needed for adequate classroom behaviors, they obtain more insight in their own learning behavior. The psycho education addresses five executive functions, based on information processing and are important in a learning situation namely: paying attention, planning skills, WM, goal-directed behavior, and metacognition. For each executive function, five sessions in the protocol are devoted to that topic. For instance in regard to paying attention, it is explained to children that sitting straight in your chair or taking a deep breath might help to focus on the task. The psycho education is offered through an audio-book, with a ‘brain castle’ metaphor. It is explained that only by following the right journey (first pay attention, make a plan, remember the task etc) in your head, i.e., ‘brain castle,’ you will manage to finish a task in the classroom. During this journey, the audio-book introduces them to the so called ‘brain guards’ (i.e., strategies such as repeat instruction or visualize) or ‘brain bandits’ (i.e., pitfalls such as distraction or acting to fast). The brain castle and it’s guards and bandits are also visualized with drawings, plastic cards and stickers. Every day the audio-book ends with a different cue (depending on which executive function is discussed), for example ‘I repeat what is said.’ This cue will be repeated throughout the session by the coach if necessary and the cue has to be practiced within a neuropsychological – and school task related exercise.

Second, this intervention contains three paper and pencil adaptive WM tasks: a visual spatial span task, a listening recall span task, and an instruction paradigm task (30 trials in total) which are practiced on a daily basis to improve WM capacity. The sequence of each trial is extended after two correct trials. In the listening recall tasks, the coach reads aloud a certain amount of sentences and the child has to evaluate and tell whether the particular sentence is true or false. After this, the child has to reproduce the last word of each sentence in the correct order. The visual spatial span task is a paradigm of the Corsi block-tapping task (Corsi, 1972) which consists of a template with ten small blocks. The child has to tap the same cubes as the coach but then in the reversed sequence. The instruction task was based on a previously described analog task (Gathercole et al., 2008) and consists of a paper template and cards that contains pictures of school related items. The coach reads aloud an instruction that the child has to execute for example “*Point to the big circle and pickup the small blue pen.*” For each next level one action or one extra item was added so the next sentence could be “*Pickup the large yellow book and a scissor and put them on the small square.*” Each WM task was ended after ten executed trials. At the end of each session, the child fills out a high score list for each task to keep track of their performance.

The third key element of this intervention is the central role of optimizing generalization to the classroom-situation. First of all, the strategies and pitfalls introduced through the audio-book described above will be illustrated and practiced by performing school related tasks, such as arithmetic, in a workbook during the session. The coach stimulates the child to use the cue from the audio-book and the coach also monitors whether the child uses any of the ‘brain guards’ or whether the child encounters ‘brain bandits.’ Performance on these school related tasks is not important, in stead reflection on the process is stimulated by the coach. The second way to improve generalization to the classroom is realized by a registration card which the child brings back to class. This card contains the cue of the day (for example, ‘I repeat what is said’) and is meant to remember the child to practice the cue in the classroom. It will also inform the teacher about the cue so that he/she can monitor or stimulate the child to practice. Finally, we closely involved the teacher in the process by informing him/her with the protocol and by giving him/her an active part in the process. Teachers received a written manual, which contained information about how to recognize WM problems in the classroom and information about the intervention itself. Furthermore, they were asked to daily record whether the child applied the cue in class through structured observation forms. The structured observation forms contained four specific statements, for instance ‘The child is able to repeat the instruction,’ that had to be rated on a four point Likert scale. Subsequently, the coach reviewed this observation form the next day which gave the coach information whether the child visibly applied the cue in the classroom.

#### Standardization Interventions

Developmental psychologists were trained as ‘training aides’ according to the CWMT protocol (Gerrits et al., 2012) and also trained as therapists for the PAC intervention. During an interactive 3 h course, provided by a member of the research team, the developmental psychologists were introduced in the theoretical background and practical implications of both interventions. The PAC intervention consists of a written manual for the trainer with clear instructions for each task/component and daily score sheets for the WM tasks. Since the psychologists trained both children in the CMWT group as children in the ‘PAC’ group, they were asked not teach the specific ‘PAC’ skills to the children (i.e., not apply the psycho education) in the CWMT group. A total of 31 psychologists and five CWMT coaches were deployed in this study.

#### Treatment Adherence

For both interventions the developmental psychologists received weekly supervision by a certified Cogmed Coach and clinical staff member of the Bascule in which they discussed the progress and clinical difficulties. Also the trainers filled out a daily diary per child for observations and special circumstances. Finally the Cogmed Training Web and the PAC workbook were used to monitor the results of the training. These three documents were used to create a checklist for evaluating treatment compliance.

### Measures

Neurocognitive assessment and academic performance were the primary outcomes of this study. Behavior in class, behavior problems and quality of life were the secondary outcome measures. Assessment took place at school in a separate room at three consecutive moments: at baseline, directly after treatment, and 6 months after treatment.

#### Compliance

For both groups, we used the number of completed training sessions and improvements on the trained tasks as a measure for compliance. Treatment compliance was defined as completing twenty or more sessions, as has been reported in previous studies (Klingberg et al., 2005). For the individuals in the CWMT group, we used the *Improvement Index* as a measure of improvements on trained tasks. This index is generated by the program and reflects the difference between the *Start Index* (mean of three best trials on days 2 and 3 of the training based on two tasks) and the *Max Index* (mean of the best three trials on the best 2 days of training based on two tasks). For the individuals in the ‘PAC’ group we reported three different improvement indexes namely a *visual spatial index*, a *listening recall index* and an *instruction index*, referring to the improvements on the three trained tasks.

#### Primary Outcomes

*Neurocognitive assessment* included tasks that measure attention (Creature Counting and Score!: Manley et al., 2004), verbal WM (Digit Span:Wechsler, 2005; Comprehension of Instruction and Word List Interference: Zijlstra et al., 2010), visual spatial WM (Span Board: Wechsler and Naglier, 2008), planning skills (Six Part test BADS-C: Tjeenk-Kalff and Krabbendam, 2006), and inhibition (Inhibition: Zijlstra et al., 2010). Finally, parents and teachers filled out the Dutch version of ‘The Behavior Rating of Executive Functions’ (BRIEF) questionnaire (Smidts and Huizinga, 2009). This questionnaire consists of 75 items which can chart the following executive functions: inhibition, shifting, emotional control, initiation, WM, planning and organization, organization of materials and monitoring. These clinical scales form two broader indexes: the Behavioral Regulation Index (i.e., the scales Inhibit, Shift and Emotional Control) and the Metacognition Index (i.e., the scales Initiate, WM, Plan/Organize, Organization of Materials, and Monitor). An overall score, the Global Executive Composite, can also be calculated. T-scores of 65 and above are considered as a clinical score.

*Academic performance* was measured with tests for word reading fluency, automated math and spelling. Word reading fluency was measured with the ‘*Een Minuut Test’* (Brus and Voeten, 1973), this test consists two parallel cards which each hold 116 words. The child receives the instruction to read out loud (fast and accurate) as many as possible words in 1 min. The ‘*TempoTest Automatiseren’* (De Vos, 2010) was used to measure the degree of automated math. The test consists of four subtests: addition, subtraction, multiplication and division calculations. For each subtest, the child has to make as many as possible sums in 2 min with a maximum of 50. The *‘PI dictee’* (Geelhoed and Reitsma, 1999) was used to measure spelling skills and consists of two parallel versions (A and B). Each version consists of 135 words that are divided in nine blocks of 15 words each. For each word, a sentence is read aloud and the child is asked to write down the repeated word. From a time-saving point of view, not all blocks were administered. The starting point was the educational age of the child and if there were three or more mistakes in that block, the previous block was also administered. The test was ended if the child made eight or more mistakes in one block. All raw scores were converted into a Learning Efficiency Quotient (educational age equivalent divided by the educational age) which allows for comparison across grade and age. We also performed secondary analysis in terms of accuracy (% correct) for the word reading fluency and automated math task as these tasks had a time restriction. We calculated an accuracy score for each point in time by dividing the raw scores of correct answers through the raw scores of total amount of produced words or sums and multiplying this answer by 100. As we had no Learning Efficiency Quotient scores for these raw scores, we added a variable ‘age at assessment’ as a covariate in the modal for analysis.

#### Secondary Outcomes

*Behavior in class* was reported by the teacher using the Learning Condition Test: this is a 70 item questionnaire that measures Direct Learning Conditions (concentration, motivation, work rate, task orientation, working according to a plan, persistency) and Indirect (social orientation, social position in class and relationship with peers and teacher) Learning Conditions (Scholte and van der Ploeg, 2009). Items can be rated on a five point Likert scale, a high score indicates a negative prognoses.

*Behavior problems* were assessed by both teacher and parents using ‘The Child Behavior Checklist for Ages 6–18’ (Verhulst et al., 1996) and ‘Teacher’s Report Form for Ages 6–18’ (Verhulst et al., 1997). We reported the scale ‘Attention Problems’ since improved attention is one of the putative transfer effects of WM training; a T-score of 65 and above is considered as problematic. We also reported the scale ‘Externalizing Problems’ which consist of the two problem-scales rule breaking behavior and aggressive behavior; a T-score of 60 is considered as problematic.

*Quality of Life* was measured with the Dutch translation of the Kidscreen-27 questionnaire (Ravens-Sieberer et al., 2007) and was completed by parents and the child. It covers five dimensions of quality of life: physical well-being, psychological well-being, autonomy and parents relations, social support and peers and school environment. The raw scores are converted into T-scores: a higher score reflects a higher quality of life.

### Procedure

The ethics approval for this study was obtained from the Medical Ethical Committee (2011_269) at the Academic Medical Centre in Amsterdam, the Netherlands. After enrollment children were randomly allocated to either the Cogmed Working Memory Training or the experimental PAC intervention by a researcher independent of the research team. The Clinical Research Unit of the Academic Medical Centre composed a randomization list, stratified by age (8–10 and 11–12 years) with a block size of six. The independent researcher assigned the children in predetermined random order and 1:1 allocation. Subsequently, the independent researcher informed the training aides and Cogmed coach about the allocated condition for each child. Parents and teachers were not explicitly informed about the allocation, however, the interventions were so dissimilar in appearance and application that parents and teachers cannot be marked as blind raters. Prior to treatment they were invited to participate in an information meeting at school where they were informed about the contents of the interventions. Two to three weeks prior to treatment, parents and teachers received the questionnaires mentioned above via e-mail or hard copy on request. One week prior to treatment, a member of the research team (who was blind for the allocation) administered the neuropsychological tasks from each child at a silent (if available) room at school. Post-treatment assessment took place within 1 week after the last training session and follow-up assessment took place after 6 months. The treatment sessions were completed during morning school hours, aligned with teachers, for both intervention groups. Training periods were planned in between school holidays so that training sessions would not be interrupted for a longer period of time. Children in both intervention groups received daily small reward such as stickers or extra playtime from the coach. In addition, they received a small presents (e.g., pencil or toy) after each week of training, regardless their improvements in trained tasks.

### Statistical Methods

The intention-to-treat (ITT) approach was used to compare treatment effects. The Statistical Package for Social Sciences, version 19 (IBM SPSS 19), was used for the statistical analysis. Demographic and clinical characteristics were analyzed with independent *t*-tests for continuous variables and Chi-square and Fisher exact tests for dichotomous variables. Outliers were removed if they had a z-score of < -3.29 or >3.29 and were replaced with the second highest value. A linear mixed model was used for each outcome variable as a function of Time, Condition and Time-by-Condition interaction. Secondary analyses were performed with age and gender as covariates. Missing data was considered missing at random and was not imputed because using linear mixed model analyses has the benefit of using every observation for each participant if a baseline score is present. The covariance type for each outcome measure was based on the smallest Akaike’s Information Criterion. The significance level was set at *p* = 0.05 (two-tailed). A Bonferroni correction was performed to evaluate the effect of multiple testing which resulted in a significance level of *p* = 0.003 for the neurocognitive outcome measures (*n* = 15) and a significance level of *p* = 0.005 for the academic performance measures (*n* = 11). In addition to these analyses, Cohen’s *d* was calculated as an effect size by subtracting the difference between groups for the change scores (post – baseline and follow up – baseline for both groups), dividing that by the pooled standard deviations of both groups at baseline. A paired samples *t-*test was conducted on the mean scores of the Start- and Max Index to test whether the children in the CWMT improved significantly on the improvement index. Paired samples *t-*tests were also conducted for the *visual spatial index, listening recall index*, and *instruction index* for the children in the PAC group. Independent *t*-tests at baseline showed that groups did not differ on any of the outcome measures prior to treatment, however, there was a trend for Spelling *p* = 0.057 possibly due to the fact that were almost twice as much children with Dyslexia in the ‘PAC’ condition. The difference in Dyslexia between the two groups was non-significant however.

## Results

**FIGURE 1:**
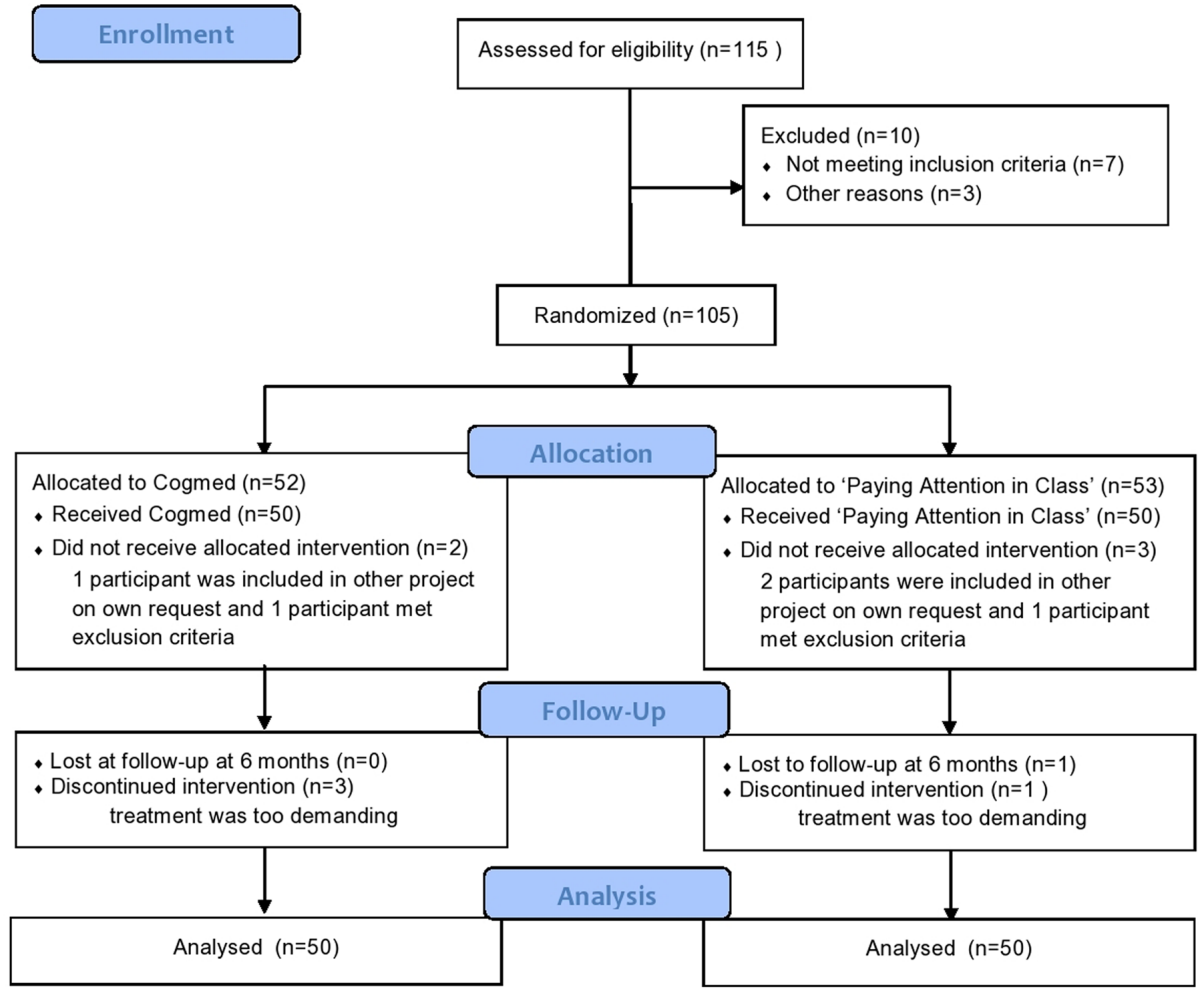
**CONSERT flow diagram**.

Between January 2012 and May 2013, a total of 115 children were assessed for eligibility; 10 children were excluded because they did not meet inclusion criteria or for other reasons (**Figure 1**). One hundred and five children were included and randomized, 52 children were allocated to the CWMT and 53 were allocated to the PAC intervention. Three children from the PAC intervention group and two children from the CWMT group did not start treatment after allocation because either they met exclusion criteria after all or they were included in a different research project due to time scheduling problems. This resulted in 50 children starting with CWMT and 50 children starting with PAC.

### Compliance Measures

Of the 50 children who followed CWMT, 47 children (94%) met the compliance criteria of 20 or more complete sessions. Paired samples *t*-test showed that children in the CWMT group improved significantly on the *Improvement Index* with a mean *Max Index* of 94.25 (SD = 12.71) and a mean *Start Index* of 72.62 (SD = 9.26), *t*(49) = -17.796, *p* < 0.001. Of the 50 children who followed the PAC training, 46 workbooks were available for analysis of compliance. Forty-two children (91.3%) met the compliance criteria of twenty or more complete sessions (i.e., psycho education, tasks in workbook, and WM tasks). Paired samples *t*-test showed that children improved significantly on the *visual spatial index* with a mean of 3.5 (SD = 0.74) at the start of training and a mean of 5.42 (SD = 1.35) at the end of training, *t*(47) = 11.409, *p* < 0.001. Children also improved significantly on the *listening recall index* with a mean of 2.45 (SD = 0.72) at the start of training and a mean of 4.40 (SD = 1.21) at the end of training, *t*(46) = 11.758, *p* < 0.001. Finally, children improved significantly on the *instruction index* with a mean of 3.54 (SD = 1.01) at the start of training and a mean of 8.29 (SD = 1.96) at the end of training, *t*(47) = 18.24, *p* < 0.001.

### Primary Outcomes

#### Neurocognitive Assessment

As can be seen in **Table 2**, a significant effect of time at post-treatment was found for attention (Creature Counting, correct answers; *p* = 0.000), verbal WM (Word List Interference Remember; *p* = 0.000, Comprehension of Instruction; *p* = 0.000), visual spatial WM (Span Board; *p* = 0.000), inhibition (Inhibition correct answers; *p* = 0.000 and time; *p* = 0.000), parent rated Behavioral Regulation Index (*p* = 0.003) and Metacognition Index (*p* = 0.000). A significant effect of time at post-treatment for Score! (sustained attention) was also found, however, this was a decrease.

**Table 2:** Results on neurocognitive assessment.

	Baseline		Post-treatment		Follow-up		*p* Effect time pre–post	*p* Effect time pre-fu	*p* Effect group	*p* Interaction effect	*d^1^* (CWMT-PAC)	*d^2^* (CWMT-PAC)
	CWMT	PAC	CWMT	PAC	CWMT	PAC						
Score!	8.7	8.2	7.8	6.8	9.6	8.6	0.000^a^	0.137	0.06	0.537	0.19	0.19
Creature counting							
Correct	9.3	9.6	11.3	10.9	10.8	10.1	0.000^a^	0.013^b^	0.372	0.346	0.26	0.38
Time	9.5	9.8	10.3	9.3	10.9	10.4	1	0.015^b^	0.448	0.151	0.38	0.23
Digit Span	9.5	8.8	11.2	8.8	10.7	9.2	0.021^b^	0.004^b^	0.009^b^	0.018^b^	0.57	0.27
Span board	47.7	45.3	58.8	48.2	56.3	49.1	0.000^a^	0.000^a^	0.000^a^	0.000^a^	0.85	0.49
WLI							
Repeat	9.9	9.9	10.3	10.6	10.6	10.5	0.039^b^	0.005^b^	0.919	0.666	-0.13	0.04
Remember	11.7	11.1	13	13.2	12.4	12.7	0.000^a^	0.000^a^	0.947	0.12	-0.33	-0.13
Six part test	8.8	8.9	9.7	9.9	10.5	10.2	0.012^b^	0.000^a^	0.926	0.729	-0.04	0.14
COI	9.3	9.2	11	11.1	11.1	10.8	0.000^a^	0.000^a^	0.824	0.728	-0.08	0.08
Inhibition switching							
Mistakes	7.5	7.5	5.4	4.9	4.7	5.6	0.000^a^	0.000^a^	0.811	0.137	-0.09	0.16
Time	113.2	111	101.9	98.6	94.6	94.3	0.000^a^	0.000^a^	0.595	0.522	-0.02	0.1
BRIEF parents							
BRI	56.1	54.6	53.8	52.8	55	54	0.003^a^	0.606	0.46	0.93	-0.05	-0.05
MCI	59.7	61	56.6	57.8	57.9	58.8	0.000^a^	0.033^b^	0.494	0.973	0.01	0.05
BRIEF teacher							
BRI	63.5	60.3	63.3	57.8	58.6	58	0.85	0.102	0.217	0.379	0.14	-0.16
MCI	67.1	67.2	63.4	64.9	60.1	61.8	0.019^b^	0.003^a^	0.682	0.811	-0.07	-0.09

*CWMT, Cogmed Working Memory Training; PAC, Paying Attention in Class; WLI, word list interference; COI, comprehension of instruction; BRIEF, Behavior Rating of Executive Functions; BRI, Behavioral Regulation Index; MCI, Metacognition Index. Raw scores where used for amount of correct answers and time for the Inhibition task; Span board and BRIEF scores are expressed in *t*-scores; all other scores are expressed in standard scores. *d^1^* = difference between groups for the change scores post to baseline for both groups, divided by the pooled standard deviations of both groups at baseline. *d^2^* = difference between groups for the change scores follow up to baseline for both groups, divided by the pooled standard deviations of both groups at baseline.*

*^a^*p* = <0.003 (significant after Bonferroni correction); ^b^*p* < 0.05.*

At follow-up, significant effects of time were found for verbal WM (Word List Interference Remember; *p* = 0.000, Comprehension of Instruction; *p* = 0.000), visual spatial WM (Span Board, *p* = 0.000), planning (Six Part test; *p* = 0.000), inhibition (Inhibition correct answers; *p* = 0.000 and time; *p* = 0.000) and teacher rated Metacognition Index (*p* = 0.003).

A significant group effect was found for the Span Board task (*p* = 0.000, *d*^1^ = 0.87; *d*^2^ = 0.49) in favor of CWMT. An interaction effect was also found for Span Board task (*p* = 0.000). When the forward and backward condition for Span Board task were analyzed separately, results showed that there was only a significant group (*p* = 0.000) and interaction (*p* = 0.000) effect for the Forward condition.

#### Academic Performance

There were no significant time, group or interaction effects on the Learning Efficiency Quotient scores of word reading fluency (**Table 3**). Results showed one effect of time at follow up for the subtest ‘division’ of the automated math task (*p* = 0.005), however, this was a decrease of performance. It should be noted here that sample size of the multiplication and division subtests at baseline was a lot smaller than the sample size of the multiplication and division subtests at follow up. The subtests multiplication and division were not administered for children in lower grades as they do not yet acquire these multiplication and division skills yet. After Bonferroni correction results revealed a trend group effect (*p* = 0.036) and trend effect of time at follow up (*p* = 0.045) for spelling. As children in the CWMT group already performed better at baseline, we suspected that Dyslexia moderated the results. When Dyslexia was entered in the model as a covariate, the trend effect of group was no longer present (*p* = 0.150).

**Table 3:** Learning efficiency quotients of academic performance measures.

	Baseline		Post-treatment		Follow-up		*p* Effect time pre–post	*p* Effect time pre-fu	*p* Effect group	*p* Interaction effect	*d^1^* (CWMT-PAC)	*d^2^* (CWMT-PAC)
	CWMT	PAC	CWMT	PAC	CWMT	PAC						
WRF	0.783	0.731	0.761	0.695	0.854	0.755	0.614	0.105	0.276	0.451	0.04	0.15
Automated math							
Addition	0.729	0.672	0.736	0.701	0.717	0.675	1	1	0.413	0.855	-0.07	-0.05
Subtraction	0.658	0.636	0.688	0.628	0.662	0.609	1	1	0.375	0.573	0.14	0.11
Multiplication	0.761	0.765	0.798	0.802	0.716	0.743	0.614	1	0.872	0.952	0	-0.01
Division	0.693	0.714	0.719	0.715	0.639	0.646	1	0.005^a^	0.863	0.766	0.1	0.06
Spelling	0.692	0.584	0.723	0.608	0.756	0.625	0.238	0.045^b^	0.036^b^	0.856	0.03	0.11

*CWMT, Cogmed Working Memory Training; PAC, Paying Attention in Class; WRF, Word reading fluency. All scores are expresses in a learning efficiency quotient. *d^1^* = difference between groups for the change scores post to baseline for both groups, divided by the pooled standard deviations of both groups at baseline. *d^2^* = difference between groups for the change scores follow up to baseline for both groups, divided by the pooled standard deviations of both groups at baseline.*

*^a^*p* = < 0.005 (significant after Bonferroni correction); ^b^*p* < 0.05.*

For the accuracy scores (see **Table 4**) results showed a significant group effect in favor of CWMT (*p* = 0.003) on word reading fluency, but without a significant interaction effect (*p* = 0.312). Further inspection of the data revealed that children from the CWMT group already significantly performed better at baseline (*p* = 0.004) than the children in the ‘PAC’ group possibly due to the fact that were almost twice as much children with Dyslexia in the ‘PAC’ condition. We therefore again entered Dyslexia as a covariate in the model and found that the group effect was no longer significant (*p* = 0.046) after Bonferroni correction. Finally, we found no significant time, group or interactions effects for the accuracy scores of the automated math task.

**Table 4:** Accuracy scores of Word reading fluency and Automated math.

	Baseline		Post-treatment		Follow-up		*p* Effect time pre–post	*p* Effect time pre-fu	*p* Effect group	*p* Interaction effect	*d^1^* (CWMT-PAC)	*d^2^* (CWMT-PAC)
	CWMT	PAC	CWMT	PAC	CWMT	PAC						
WRF	96.4	93.2	96.4	94.3	97.2	95	0.952	0.584	0.003^a^	0.306	-0.21	0.13
Automated math					
Addition	96.4	96.7	97.4	97	96.3	97.2	0.652	1	0.709	0.499	0.13	-0.11
Subtraction	91.7	93.5	95.1	92	93.9	93.5	1	1	0.614	0.055	0.15	0.17
Multiplication	91.7	90.2	91.3	89.6	92.9	93.2	1	0.869	0.555	0.574	0.02	-0.08
Division	88.8	86.5	86.5	84	90.5	91.6	1	0.936	0.691	0.537	0.01	-0.17

*CWMT, Cogmed Working Memory Training; PAC, Paying Attention in Class; WRF, word reading fluency. All scores are reflect the percentage of correct answers. *d^1^* = difference between groups for the change scores post to baseline for both groups, divided by the pooled standard deviations of both groups at baseline. *d^2^* = difference between groups for the change scores follow up to baseline for both groups, divided by the pooled standard deviation of both groups at baseline.*

*^a^*p* = < 0.005 (significant after Bonferroni correction).*

### Secondary Outcomes

#### Behavior in Class

Analyses for the Direct Learning Condition scale showed no significant effects of time (post-treatment; *p* = 0.395, follow-up; *p* = 1.000), group (*p* = 0.060), or interaction (*p* = 0.068). Non-parametrical tests were performed for the Indirect Learning Conditions scale since data was not equally distributed. We only found a significant decrease for the CWMT group from pre treatment (*M* = 60.23) to follow-up (*M* = 57.27), *p =* 0.022. However, this decrease was not significantly different from the PAC group (*p* = 0.975).

#### Behavior Problems

Parent ratings of ‘Attention Problems’ showed a significant effect of time at post-treatment (*p* = 0.000) and follow-up (*p* = 0.000). There was no significant group (*p* = 0.593) or interaction effect (*p* = 0.138). The parent rated scale of ‘Externalizing Problems’ also showed a significant effect of time at post-treatment (*p* = 0.000) and follow-up (*p* = 0.000) but no significant group (*p* = 0.627) or interaction effect (*p* = 0.243). Teacher rated ‘Attention Problems’ also showed a significant effect of time at post-treatment (*p* = 0.007) and follow-up (*p* = 0.001) but no significant group (*p* = 0.149) or interaction effect (*p* = 0.558). No significant time, group, or interaction effect was found for the scale ‘Externalizing Problems’ as rated by teachers.

#### Quality of Life

We found no significant time, group or interaction effects for any of the five dimensions of quality of life that were rated by parents or the child.

## Discussion

The aim of this study was to replicate and extend previous studies of CWMT in school-aged children with ADHD. This was the first randomized controlled trial that contained an active control group in which children received adaptive WM tasks in response to performance, equal interaction time with the coach and performance related feedback. Therefore, in contrast to previous effect studies of CWMT in children with ADHD, the experiences of the trained and control group were more similar in terms of effort and expectations in current study. Another strong aspect of current study was the fact that, next to broad neurocognitive measures, it included long term (6 months) assessments of areas that reflect functioning in everyday life, i.e., academic performance, behavior in class, behavior problems, and quality of life in a noteworthy large sample.

Although results showed an effect of time on verbal WM, attention, inhibition, planning, parent, and teacher ratings of executive functioning and ADHD related behavior, no superior effect of CWMT was found on these measurements in comparison to the effects of the PAC intervention. No significant time or treatment effects were found for academic performance, behavior in class, and quality of life. We were only able to replicate one treatment effect on visual spatial WM as was also found by previous efficacy studies of CWMT in children with ADHD (Klingberg et al., 2002, 2005; Gray et al., 2012; Hovik et al., 2013). Our results showed that the treatment and interaction effect was only apparent for the Forward condition of the Spatial Span task which suggests that CWMT only had a superior effect on short term memory in comparison to the PAC intervention, as was previously pointed out by Rapport et al. (2013). Most trained tasks within CWMT contain visual spatial (working) memory elements which strongly resembles the Spatial Span task that was used for the assessment of visual spatial WM. In contrast, the PAC intervention contains only one trained task that resembles the Spatial Span task. Therefore we suggest that this treatment effect should be viewed as a practice effect and not a measure of (near) transfer. We were not able to replicate treatment effects that were previously found on verbal WM (Holmes et al., 2010; Hovik et al., 2013), measures of attention (Klingberg et al., 2002, 2005; Egeland et al., 2013), parent ratings of ADHD (Klingberg et al., 2005; Beck et al., 2010) and executive functioning (Beck et al., 2010) and measures of academic performance (Green et al., 2012; Egeland et al., 2013). We suggest that there are several explanations for the fact that current study could not replicate treatment effects of CWMT that were found in previous studies.

First of all, regarding the neurocognitive measures, we suggest that the difference in control groups added to these inconsistencies. For instance, previous studies have used no-contact control groups such as treatment as usual (Egeland et al., 2013; Hovik et al., 2013) which corrects for test–retest effects. However, it does leave the possibility open that the trained and control group approached the post-assessment differently in terms of motivation (Shipstead et al., 2012a). This same argument also accounts for the studies that used low-demand, non-adaptive control groups (Klingberg et al., 2002, 2005). Improvements on post-training measures might reflect the belief that training should have a positive influence on cognition (Morrison and Chein, 2011). It is questionable whether the use of a low-demand, non-adaptive control group sufficiently convinces participants that they are engaged in cognitive training (Shipstead et al., 2012a). As results did indicate effects of time, we suggest that non-specific treatment factors partially might explain current findings. We suggest that positive reinforcement during training should be considered as a plausible mechanism. Next to models that view executive dysfunction as a causal model for ADHD, there are also models that emphasize the sub-optimal reward systems (delay aversion/motivational style) as a second and co-occurring causality for ADHD (Sonuga-Barke, 2003). Dovis et al. (2012) showed that incentives significantly improved WM performance of children with ADHD and the intensity of the incentive determined the persistence of performance over time. In our study, children in both groups received performance related feedback during training and were encouraged during performance. In addition, they received daily small reward at the end of each session (e.g., stickers or playtime) and a small present on a weekly basis. It is plausible that the encouragements and incentives obtained during training altered their motivation in regard to performance. Despite the strong design of current study, it should be noted that this study did not contain a ‘no treatment’ control group (e.g., waiting list) as a third arm for allocation. Therefore we cannot rule out other possible cofounders such as test–retest effects, passage of time or therapeutic benefit. Choosing and developing control groups remains challenging for future trials as ethical constraints make it difficult to implement ‘no treatment’ groups and there still is no consensus about how a control group should be designed (von Bastian and Oberauer, 2014).

Regarding the results on academic outcome measures, we suggest that the heterogeneity of the used samples make it difficult to interpret results across CWMT studies. For instance, while current study included both inattentive and combined subtype children, others (Egeland et al., 2013) only included children with the combined subtype. Another factor that could contribute to the inconsistencies in results concerns the inclusion of children with comorbid learning difficulties. For instance, just as current study, Gray et al. (2012) used a sample of children with comorbid LDs, others (Egeland et al., 2013; Chacko et al., 2014) did not report whether they included children with comorbid learning difficulties. Recently it has been suggested (Sonuga-Barke et al., 2014) that the response to different forms of training should be compared between clinical subtypes and neuropsychological subgroups. Furthermore, we suggest that future research should pay closer attention to individual differences such as age, biological factors, personality, and initial cognitive ability as these factors have been mentioned as potential moderators of treatment effect (Jaeggi et al., 2011; Jolles and Crone, 2012; von Bastian and Oberauer, 2014). For instance, it was suggested that WM training might be more effective for subgroups of ADHD, for instance ADHD plus WM problems (Chacko et al., 2013). This would reflect the ‘room for improvement’ hypothesis in which children with a lower ability at the start of training (for instance WM) show larger improvement on training gains as there would be more room for improvement than children with more normal ability levels who will reach their ceiling capacity much faster. A study of Holmes et al. (2009) might support this view as they showed that mathematical ability improved in children with low WM skills after following WM training.

Next to paying more attention to individual differences, we also suggest, in line with current comments of Gathercole (2014), that future research should take a closer look into how to assess academic performance. Many previous studies contained standardized ability tests for complex skill domains such as reading and mathematics. According to Gathercole (2014) the problem with these standardized ability tests is that they tap cumulative achievements which makes them strongly dependent on prior learning and relatively insensitive to recent changes in learning capacities. Determining the true and distinctive effect of training in terms of academic outcome measures remains challenging as there is one complicating factor that is often overlooked. While test–retest effects and maturation (passage of time) are often taken into account, it is much harder to control for the potential new skills that children have been exposed to in between assessment periods. In addition, children in lower grades are most likely more frequently exposed to new skills during a certain time period in comparison to children in higher grades. One possible way to overcome this problem is by following the example of a study from Holmes and Gathercole (2014). They used National Curriculum assessments in English and math to calculate the sublevel improvements for the relevant academic year. Conclusively, despite the fact that our results are in line with most recent meta-analyses (Rapport et al., 2013; Cortese et al., 2015), we suggest that more information can be gained from future trials if individual differences and solid academic outcomes measures are taken into account.

Finally, regarding the effects on parent and teachers ratings of ADHD related behavior and executive functioning, we again suggest that the difference in control groups added to the inability to replicate treatment effects of previous CWMT studies. It has been previously suggested that non-adaptive placebo control interventions (e.g., Klingberg et al., 2005) require considerably less time and effort from the coach (usually parent) than active conditions. This has direct implications for interpreting parent-rated improvements as it diminishes the quantity and quality of parent-child interaction (Chacko et al., 2013). Also, studies that used non-active (e.g., waiting list, treatment as usual) control groups (Beck et al., 2010) might have created bias as these type of control groups hinder blinding (Sonuga-Barke et al., 2014). It is possible that post-test change may reflect expectations that were created by the act of receiving treatment rather than actual changes that were brought about by treatment (Morrison and Chein, 2011; Shipstead et al., 2012b). In current study, parents were not involved in the delivery of the interventions and the interaction time with the coach was equal for children in both groups. Therefore, we suggest that treatment effects on parent ratings of ADHD (Klingberg et al., 2005; Beck et al., 2010) and executive functioning (Beck et al., 2010) in previous studies should be interpreted with caution. However, although parents were not actively informed about treatment allocation in current study, they cannot be considered objective raters as it was communicated that both interventions were active. A meta-analysis of Sonuga-Barke et al. (2013) showed that effects of ADHD ratings after cognitive interventions dropped to non-significant if outcomes of probably blinded raters were considered. This same argument might also explain current effects of time on teacher ratings. Both interventions were delivered at school during school hours so teachers were daily reminded that children were receiving treatment. Furthermore, teachers were invited to attend an information meeting that contained information about WM problems in the classroom and information about the interventions. From a clinical perspective, we can only encourage the involvement of teachers in such intensive interventions. However, from a scientific point of view it remains challenging how to incorporate teachers perspective. We suggest that future studies should incorporate classroom observation rated by blinded and objective persons. As was suggested by Green et al. (2012), teachers are probably less objective as they already formed a general impression of the behavior patterns of a child and they may not be sensitive in detecting positive changes.

## Conclusion

In summary, when compared to an active intervention, a superior effect of CWMT could only be found on a trained visual spatial WM task. Although children in both groups improved on broad measures of neurocognitive functioning supported by both parent and teacher ratings, these results should be interpreted with caution as they might be related to methodological and non-specific treatment factors. We suggest that future trials with well-blinded measures, a third ‘no treatment’ control group and adequate (far) transfer measures are needed before cognitive training can be supported as an evidence-based treatment of ADHD. Furthermore, we suggest that future studies should be aimed at gaining more insight in *why* and *how* cognitive training is effective with possible support from neuro-imaging studies. This might shed some light on the question why some of the transfer measures are improved and others are not and may subsequently lead to improved intervention designs. Another important area to explore regards the area of *who* could benefit most from cognitive training. This concern would be of high clinical value in terms of treatment adherence, financial resources and effort resources from children, parents, teachers, and health care professionals.

## Conflict of Interest Statement

The authors declare that the research was conducted in the absence of any commercial or financial relationships that could be construed as a potential conflict of interest.
